# Exploring what matters to residents of Australian aged care facilities with the Happy Life Index: comparison of qualitative responses between pre- and mid-Covid-19 pandemic time points

**DOI:** 10.1007/s11136-023-03387-0

**Published:** 2023-03-16

**Authors:** Carolyn M. Murray, Steve Milanese, Michelle Guerin, Rebecca Bilton, Katherine L. Baldock, Gaynor Parfitt

**Affiliations:** 1 International Centre for Allied Health Evidence, University of South Australia, Adelaide, Australia; 2 Allied Health and Human Performance Unit, University of South Australia, Adelaide, Australia; 3 Alliance for Research in Exercise, Nutrition and Activity, University of South Australia, Adelaide, Australia; 4 IIMPACT in Health, University of South Australia, Adelaide, Australia

**Keywords:** Health services, Elderly, Satisfaction, Lifestyle, Staffing, Feedback, Social interactions

## Abstract

**Purpose:**

This study analysed data from a national survey of people living in Australian Residential Aged Care Facilities (RACFs) reporting on what is the best thing about where they live and suggestions for improvement. Data from prior to the Covid-19 pandemic were compared with data during the Covid-19 pandemic.

**Methods:**

Qualitative data from the Happy Life Index Survey were analysed using summative content analysis to code the responses in the data sets and then organise them into categories. Once categorised, the pre-Covid-19 and mid-Covid-19 data sets were compared using descriptive statistics.

**Results:**

A total of 4745 residents, from over 100 RACFs, provided 8512 open-text responses to at least one of the two survey questions. Pre-Covid-19 responses were compared with mid-Covid-19 responses and those trending towards relevance (5–10% change) were identified. There were both positive and negative relevant percent changes for staff number, food (general comments), and friendliness. A trending positive percentage change was observed for staff quality and the internal environment. There was a trending negative relevant percentage change for lifestyle activities, staff generally, level of contentedness, the general environment, general choice, and general views about the service.

**Conclusion:**

People living in RACFs notice the changes in staffing levels and visitors during restrictions imposed during infectious outbreaks. During these times, they appreciate the quality of the staff attending to their needs and the quality of their food. Further exploration is needed of the value of lifestyle activities and strategies to promote feelings of contentedness and general wellbeing during times of restriction.

## Plain English summary

People living in residential aged care settings receive support with daily living tasks, social interactions and activities that maintain quality of life. External monitoring of resident satisfaction with facilities provides opportunity for service maintenance and feedback. This feedback process occurs in some Australian facilities using the Happy Life Index administered by CarePage Ltd. This tool includes two open questions asking what the best thing about the facility is, and what is an area for improvement. This study sought to compare the responses at two time points (before the Covid-19 lockdowns and restrictions and afterwards). We found that during times of restrictions, the staffing levels and visitor frequency was noticed by residents, with quality of food and staff mattering the most. More exploration is needed about the value of lifestyle activities and strategies for continuation of visitors during times of restrictions.

## Introduction

The rise in antibiotic-resistant bacterial infections in the community, an increasing national rate of known threats such as influenza and whooping cough, and the increasing emergence of new diseases, such as novel Middle East respiratory syndrome coronavirus (MERS-CoV) and avian influenza (H5N1 and H7N9), have made the control of communicable diseases a national priority [1]. Older persons are at an increased risk of communicable diseases, due to their reduced immune function, frailty, and the increased presence of comorbidities [2]. People living in residential aged care facilities (RACFs) are a particularly vulnerable group of older persons, secondary to the sustained proximity with other residents, and exposure to external visitors, including staff who may be working across multiple sites [3, 4]. RACFs are “special-purpose facilities which provides accommodation and other types of support, including assistance with day-to-day living, intensive forms of care, and assistance towards independent living, to frail and aged residents” [5]. They are also known as long-term care, care homes, nursing homes, and skilled nursing facilities. In Australia, RACFs can be government run or privately owned but all must be accredited by the Aged Care Standards and Accreditation Agency Ltd to receive funding from the Australian Government through residential aged care subsidies.

Restrictions of RACFs to visitors, limits on intra-facility movement, and limiting of staff to one site are some of the control mechanisms used for risk mitigation to prevent infectious disease transmission in an environment where residents live in proximity. Whilst the widespread use of lockdowns by governments during the current Covid-19 pandemic has been seen as controversial by some, they were used in RACFs prior to Covid-19 to help control other outbreaks, such as influenza [6].

On 18 March 2020, the Australian Federal government announced a raft of directives for RACFs including restrictions on visitor access, the need for social distancing, and the use of personal protective equipment to help stop the spread of Covid-19 [7]. This statement advised RACFs to limit visits from family and friends, particularly for residents with chronic illness. Following the implementation of these restrictions, the individual states instigated programs of lockdowns to help control the community spread of Covid-19. These lockdown practices in the aged care sector in Australia, and isolating RACFs early, may have contributed to the lower death rate among people living in RACFs in Australia compared to the UK [8], who were slower with instigating restrictions. Lockdowns or visitor restrictions in RACFs have become a widespread intervention globally to halt or slow the spread of Covid-19 among this vulnerable sector of the community [9].

With the rise of lockdowns and visitor restrictions in RACFs came concerns about the risk of social isolation and loneliness among residents with complex medical issues [10]. There is a growing body of research specific to the Covid-19 pandemic that has shown connections between decreased quality of life and community lockdowns [11]. This reduction in quality of life is perpetuated by social isolation and feelings of loneliness, possibly leading to negative physical and mental health among older adults [12, 13]. Specifically, the duration of isolation can be an important predictor of negative effects on mental health [14]. However, much of the existing research has occurred with people living in the community rather than living in RACFs and does not have pre-pandemic data (baseline) for comparison [15]. This creates a gap in understanding, particularly as people living in RACFs are not only subject to community lockdowns but may also experience restrictions on movement within their environment and have challenges with managing phones and technology [16]. As a result, they often depend on visitors and staff for their social interactions and are potentially at high risk of a decline in mental health during lockdowns and restrictions [17].

Prior to the pandemic, studies into the quality of life of residents in RACFs identified that sense of control and physical health were strongly related to the self-reported quality of life [18]. Other factors that have been shown to relate to resident reported quality of life include staff and resident relationships, autonomy and respect, sense of community, food, and drink, and activities [19]. In keeping with this literature, this study aimed to understand perceptions of what Australian older people living in RACFs like best about their facility and the suggested areas for improvement at two different time points: before the Covid-19 pandemic and during the Covid-19 pandemic (with the associated restrictions). This study will address the gap in knowledge about the perspectives of those living in RACFs and provide unique insight into the effects of the access restrictions put in place to mitigate risk and control the spread of Covid-19.

## Method

### Study design

The research is a non-experimental descriptive questionnaire design [20] using summative content analysis [21] and some descriptive statistics to compare response rates between the data sets. The study used secondary data collected from over 100 RACFs across five states in Australia. The objective of this analysis was to compare the open-text responses from residents in RACFs before Covid-19 with the responses given during the Covid-19 pandemic-related restrictions.

### Data source and management

The Happy Life Index (HLI) is a commercially available online consumer-completed survey tool developed by CarePage™, in conjunction with an aged care advocacy group, and the Aged Care Guild (https://happylifeindex.com.au/) [22]. The HLI is used by RACFs nationally, with the data reported back to the RACFs in real time. The HLI asks residents to score seven indicators for satisfaction and wellness (care quality, environment and cleanliness, food quality, activities, and lifestyle, staff friendliness, staff presence, and management) using a simple 0–5 scale [22]. The HLI also includes two open-text questions which ask the residents of RACFs to summarise their perception of the facility:

- What is the best thing about the home/facility?
- What can be improved in the home/facility?

The HLI tool has been developed to be completed on a tablet device through the CarePage™ survey application. This is designed with an interface that is easily understood by older adults, but the HLI also allows the resident to receive help with the completion of the survey if needed. Where this is required, the assistant is someone not involved in the direct care of the resident (i.e. admin, volunteers, students). This study explored the responses to the two open-text questions.

A pragmatic decision was made to restrict pre-Covid-19-related data to any data collected before 1 January 2020, and mid-Covid-19 responses being any data collected between 1 April 2020, and the end of July 2021. April 2020 was selected as each Australian state had its first reported Covid-19 case by then and the Australian Federal government aged care directives were enacted on 18th March 2020. From this time RACFs restricted visiting for families and friends of residents. Whilst general community lockdown restrictions began to ease, the states and territories began taking control based on health advice. However, a national survey in March 2021 of 2559 RACFs identified that over 70% of RACFs still maintained some form of restriction to visitor access [23]. We, therefore, considered it reasonable to expect that the Covid-19 restrictions would have an impact on residents from 1 April 2020, through until the middle of 2021. Any responses from 1 January 2020 to 31 March 2020 were excluded from analyses to ensure a clear differentiation between pre- and mid-Covid-19 time periods.

### Data analysis

A database of open-text responses was received from CarePage™ in MS Excel [24]. The data set was organised into two separate spreadsheets with pre-Covid-19 and mid-Covid-19 data for both questions (i.e. four separate data sheets). Initial coding [21] was undertaken, whereby a code sheet was built by two researchers (SM and GP) based on a random sample of the responses and coding was commenced by a research assistant. Following this, two researchers experienced in working with qualitative data (CM and MG) continued the analysis by iteratively adding to and adjusting the original code sheet as they worked through the four data sheets. To stay close to the data sets, all codes were closely aligned with the words used by respondents. The researchers worked on the same document meaning coding did not occur blindly but once there was a shared understanding of the process, reviewers made independent decisions about adding new or modifying existing codes.

A number was given to each code and used in the code sheet to assign responses. Responses were only assigned to one code. However, responses that referred to two codes (i.e. food and staff) were broken up and assigned accordingly. As the code sheet was built over the course of data analysis, the researchers went back to the data that were coded first to revise and update based on the new codes. This analysis included discussions to make consensus decisions about interpretation and combinations of codes. Once all coding was complete across all four code sheets the researchers rechecked for consistency and met to decide on the final categories for organising the coded data. Because there was overlap across the responses to the two questions (i.e. participants gave suggestions for improvement in response to the ‘what is best question’ and vice versa), the final categories represented the overall responses to the two questions. The pre-Covid-19 and mid-Covid-19 data sets were kept separate.

The second phase of summative content analysis [21] involved the calculation of frequencies for each code to enable comparison across the findings in the two time points. This phase of analysis was done by a third researcher with expertise in quantitative analysis (SM). The frequency of the responses for each code, calculated as a proportion of the total responses in that survey were calculated for both pre- and mid-Covid-19 surveys. The relative proportion of responses for each code across a survey reflects the relative value of that code across the survey population. Where a particular code was considered important across the survey population it was expected that the relative number of times that code appeared in responses would be greater. The proportion of responses for each code in the mid-Covid-19 survey was then subtracted from the proportion in the pre-Covid-19 survey. To minimise the chance of a type II error a pragmatic decision was made that changes greater than 10% between the pre- and mid-Covid-19 survey responses were relevant, whilst a change between 5 and 10% reflected *a trend* towards relevance. To identify if there were significant differences in the number of responses identified pre-Covid-19 compared to mid-Covid-19, data were entered into a 2 × 2 table, and chi-square analysis was performed. Statistical significance was set at *p* < 0.05.

## Results

### Participants

These responses came from 4761 residents; of these 2829 were provided before Covid-19 and 1,932 were provided during the Covid-19 pandemic in 2020. Respondents were located across New South Wales, Victoria, Queensland, South Australia, and Western Australia (Table 1). The highest number of respondents were from Victoria, closely followed by New South Wales. There were no pre-Covid-19 responses from Western Australia.

**Table 1:** Number of residents by State of residence (*n* = 4761)

State	Pre-Covid	Mid-Covid
Queensland	161	174
New South Wales	772	621
Victoria	1290	618
South Australia	606	402
Western Australia	No data	117
Total	2829	1932

### Categories and codes

The findings from the first phase of summative content analysis are shown in Table 2, which outlines the categories and codes for the content of the two open-text questions. Overall, there were 12 categories developed from the open-text questions which describe the aspects of quality reported by residents when asked openly to name areas for improvement and what is best about their RACF. Across the 12 categories, there were seven that arose logically from the data because responses reflected observational opinions of the RACFs (the first seven presented in Table 2). The remaining five were more emotive, requiring greater interpretation by the research team (the last five categories presented in Table 2). The nuances and complexities of content within each code are described in the summary column of Table 2.

**Table 2:** Categories, codes, and their descriptors

	Category	Code	Summary of content within each code
Observational opinions of RACF	Staff	Number	The number of staff in the facility
		Quality	The quality of interaction with staff i.e. friendliness; nature, approachability; genuineness
		General	Other staff-related issues, such as training, qualifications, ethnicity, language spoken
	Food and beverages	Quantity	Amount of food (i.e. size, frequency of meals/snacks)
		Quality	Quality of food (i.e. temperature, standard of meals/snacks
		General	Other food-related issues i.e. menu, timeliness, variety; choice
	Environmental	Internal	Inside facility i.e. personal room; furniture; lighting
		External	Outside facility i.e. Gardens and outside areas
		General	General environmental issues such as noise levels, pets, pest control, cleanliness, Internet access
	Lifestyle	Lifestyle activities	Activities conducted by the RACF – bus trips, exercises, concerts
	Communication	Family	Communications with family/visitors
		Staff	Communications with staff
		General	General communication issues in the facility
		Preferred language	Language related communication (non-English speaking)
		Sensory loss	Hearing loss and vision affecting communication
		Facility	Communication with management—‘having a say’
	Independence/freedom	Privacy	Level of privacy
		Shut in	Level of freedom in the facility
	Location, cost, pragmatic	Location, cost	Location/costs of RACF
Emotions/feelings-based opinions about RACF	Having needs met	ADLs	General care related to Activities of Daily Living (ADLs)
		Getting out of bed	The opportunity to get out of bed
		Health and medical	Medication, access to physio; doctor
		Timing and routine	Timing of daily routine; staff responding to call bells and attending to care needs when expected
		Personal amenities	Access to personal amenities such as hairdresser, dentist, laundry, church, coffee shop
		General choice	Choice in partaking in activities etc
	Social participation/environment	Visitors/family	Access to and interactions with family and visitors
		Staff	Interactions with staff
		Other residents	Interactions with other residents in the RACF
		Friendliness	General comments about interactions with people/environment
	Feeling secure	Safety	Level of feeling safe in the RACF
		Homely	Level of homeliness of the RACF
		Adjusting to the facility	Process of settling into the RACF
		Respect and dignity	Level of respect given to resident, including religious freedom
	General wellbeing health and mood	Mood/health	Overall mood and health
		Outlook	Overall perception of present and future
		Getting out	Appreciates getting out of their room and outside
	Views about service	General	General non-specific statements about ‘everything’ in RACF
		Level of contentedness	General comments about level of contentedness with service
		Changes in service	Recent changes in the service

### Responses to the two open questions

Given the data were organised according to the two open questions, the second phase of summative content analysis calculated the percentage change comparison between the Pre-Covid-19 responses and the Mid-Covid-19 responses to each question. There was a total of 8512 open-text responses to at least one of the two HLI open-text questions. An overview of the number of responses for each question is reported in Table 3. Some participants gave responses to both questions and some only gave responses to one question. Chi-square testing indicates that there is a significant difference between the pre- and mid-Covid-19 responses with a significantly larger proportion of responses to the best thing about their home/facility mid-Covid-19 compared to pre-Covid-19 (*p* < 0.05).

**Table 3:** Number of responses identified in each question at pre-Covid-19, and mid-Covid-19

Question	Number of responses *n* = (% of total responses)	
	Pre-covid-19	Mid-covid-19
What is the best thing about the home/facility?	1803 (38%)	2280 (61%)
What can be improved in the home/facility?	2975 (62%)	1454 (39%)

#### What is the best thing about the home/facility?

The percentage change pre-Covid-19–mid-Covid-19 for the responses to the ‘best thing’ question were calculated for each code (see Table 4). The greatest change in the mid-Covid-19 from the pre-Covid-19 responses was in the areas of ‘staff’ and ‘food’. There was an increased prevalence of reporting on the ‘quality of the staff’, but less reporting of the ‘number of staff’ as being the best thing about the facility from the resident’s perspective. General comments about the food and the quality of the food being ‘the best thing’ about the RACF declined in the mid-Covid-19 responses compared to the pre-Covid-19 responses. There was also a higher prevalence of reporting the ‘friendliness of the environment’ and the ‘safety of the facility’ in the mid-Covid-19 responses.

**Table 4:** Responses to the question “What is the best thing about the home/facility?” by code

Category	Code	Pre-Covid-19 (*n* = 1803) %	Mid-Covid-19—(*n* = 2280)	
			%	Δ from pre-Covid-19
Staff	Number	10.8	0.1	− 10.7**
	Quality	4.9	18.7	+ 13.8**
	General	5.2	8.4	+ 3.4
Food and beverages	Quantity	1.0	0.1	− 0.9
	Quality	9.4	2.1	− 7.3*
	General	12.9	2.4	− 10.5**
Environmental	Internal	5.3	8.0	+ 2.7
	External	1.2	2.2	+ 1
	General	3.1	5.6	+ 2.5
Lifestyle	Lifestyle activities	9.5	5.6	− 3.9
Communication	Family	0.3	0.0	− 0.3
	Staff	2.2	0.7	− 1.5
	General	0.6	0.0	− 0.6
	Preferred language	0.3	0.8	+ 0.5
	Sensory loss	0.1	0.1	0
	Facility	3.1	0.1	− 2.0
Independence/freedom	Privacy	0.5	2.3	+ 1.8
	Shut in	0.3	0.6	+ 0.3
Location, cost, pragmatic	Location, cost	0.3	1.6	+ 1.3
Having needs met	ADLS	2.6	1.8	− 1.2
	Health and medical	0.4	0.2	− 0.2
	Timing and routine	2.9	0.4	− 2.5
	Personal amenities	1.3	0.7	− 0.6
	General choice	1.1	1.4	+ 0.3
Social participation/environment	Visitors/family	0.1	0.8	+ 0.7
	Staff	0.1	0.2	+ 0.1
	Other residents	1.5	2.4	+ 0.9
	Friendliness	0.4	11.8	+ 11.4**
Feeling secure	Safety	0.2	6.4	+ 6.2*
	Homely	0.2	1.9	+ 1.7
	Adjusting to the facility	0.1	0.0	− 0.1
	Respect and dignity	0.1	0.2	+ 0.1
General wellbeing health and mood	Mood/health	0.0	0.1	+ 0.1
	Outlook	0.1	0.9	+ 0.8
	Getting out	0.4	0.2	− 0.2
Views about service	General	4.6	3.7	− 0.9
	Level of contentedness	11.3	7	− 4.3
	Changes in service	1.6	0.4	− 1.2

*Trending towards relevant change

**Relevant change

#### What can be improved in the home/facility?

The percentage change pre-Covid-19–mid-Covid-19 for the responses in the ‘what can be improved’ question were calculated for each code (see Table 5). Consistent with the other question, the greatest changes in prevalence of responses were in the areas of ‘food’ and ‘staff’. Table 5 shows that residents were less likely to report that improvements were required in the ‘quality of the staff’ and more likely to report that improvements in ‘number of staff’ were required during the mid-Covid-19 reporting. Residents were also more likely to report that improvements in ‘general food issues’ (timing, menu items etc.) were required. During the mid-Covid-19 reporting residents were also less likely to report that improvements were required in their ability to ‘choose to partake in activities’, the ‘friendliness of the environment’, and ‘general service’ issues. They were, however, more likely to report that improvements were required in the ‘internal environment’ of the facility.

**Table 5:** Responses to the question “What can be improved in the home/facility?” by code

Category	Code	Pre-Covid-19 *n* = 2975%	Mid-Covid-19 *n* = 1454	
			%	Δ from pre-Covid-19
Staff	Number	0.3	12.7	+ 12.5*
	Quality	23.6	8.4	− 15.2*
	General	10.1	2.0	− 8.1**
Food and beverages	Quantity	0.1	1.2	+ 1.1
	Quality	2.2	3.9	+ 1.6
	General	3.1	16.2	+ 13.0**
Environmental	Internal	2.9	10.2	+ 7.3*
	External	0.7	1.1	+ 0.4
	General	5.3	0.6	− 4.7
Lifestyle	Lifestyle activities	7.0	11.6	+ 4.6
Communication	Family	0.0	0.2	+ 0.2
	Staff	0.1	2.5	+ 2.4
	General	0.0	0.7	+ 0.7
	Preferred language	0.0	1.1	+ 1.1
	Sensory loss	0.0	0.2	+ 0.2
	Feedback pathways	1.0	1.9	+ 0.9
Independence/freedom	Privacy	1.4	0.3	− 1.0
	Shut in	1.3	1.2	− 0.1
Location, cost, pragmatic	Location, cost	0.9	0.0	− 0.9
Having needs met	ADLS	1.9	1.6	− 0.3
	Getting out of bed	0.0	0.6	+ 0.6
	Health and medical	0.0	1.2	+ 1.2
	Timing and routine	0.3	2.8	+ 2.5
	Personal amenities	0.4	1.8	+ 1.4
	General choice	6.5	0.8	− 5.7*
Social participation/environment	Visitors/family	0.2	1.2	+ 1.0
	Staff	0.1	0.2	+ 0.1
	Other residents	1.3	2.7	+ 1.4
	Friendliness	8.4	1.4	− 7.0*
Feeling secure	Safety	4.2	1.4	− 2.7
	Homely	2.1	0.1	− 1.9
	Adjusting to the facility	0.1	0.2	+ 0.1
	Respect and dignity	0.4	0.4	0.0
General wellbeing health and mood	Mood/health	0.1	0.3	+ 0.2
	Outlook	0.2	0.2	0.0
	Getting out	0.0	0.7	+ 0.7
Views about service	General	5.8	0.3	− 5.5*
	Level of contentedness	7.3	5.3	− 2.1
	Changes in service	0.5	0.8	+ 0.3

*Trending towards relevant change

**Relevant change

## Discussion

This research has analysed 8,512 open-text responses from 4,761 respondents in over 100 RACFs across five states in Australia. The data have arisen from two open-text questions in the Happy Life Index which was administered pre-Covid-19 (prior to 1st January 2020) and again mid-Covid-19 (after 1st April 2020) across most states and territories of Australia (except Australian Capital Territory, Northern Territory, and Tasmania). The two open questions in the survey asked the residents to say what they saw as the best thing about their RACF and suggestions for improvement. Overall analysis of the data set resulted in 12 categories with seven being observational and five being emotive. Not surprisingly, there were codes for dignity, privacy, and feeling safe which is consistent with qualitative research pre-Covid-19 [19, 25]. Homeliness, staff time, and flexible routines are also identified as highly valued by older people in pre-Covid-19 Australian research [26].

There were also codes related to mood, wellbeing, being offered choice, having a routine, and timely attention to needs. The most pertinent categories from comparison of the pre-Covid-19 and mid-Covid-19 data sets were food (general), staff (quality and number), environment (internal), social participation (friendliness), and feeling secure (safety). Food appears to gain increasing value during the mid-Covid-19 restrictions. Residents were more likely to report that improvements in food were required with a trend towards focus on food quality rather than quantity. This finding possibly reflects an increasing insular focus as exposure to the outside world reduces. Focussing on food service, in terms of variety and quality, ensuring adequate staffing and staff training/support and highlighting the safety precautions taken during the time of a pandemic may help maintain resident satisfaction.

Having needs met seems a logical finding as those within the RACFs will likely be focussed on whether they feel they have access to suitable medical and personal care that is delivered in a timely fashion. This finding is consistent with the growing body of literature related to ‘missed care’ which highlights issues with delays in responding to care needs such as answering bells or going to the toilet [27, 28]. These issues seem to stem from staff shortage and highly complex care needs that people living in RACFs often experience. The more specific finding in this research is the notion of having general choice about when personal care takes place, who will deliver that care and what their routine will look like. The participants identified that this choice diminished mid-Covid-19. This is likely due to staff shortages becoming exacerbated during the height of Covid-19 outbreaks [29].

Residents were more likely to report on the number of staff needing improvement mid-Covid-19 but reporting on the quality of staff remained positive. This finding aligns with a presumption that relationships with staff will hold greater value during the period of restrictions where numbers of visitors are reduced [25]. Communication with and spending time with residents becomes even more vital during visitor and activity restrictions when other ‘usual’ activities that boost mood are not occurring. Furthermore, communication with those who are unable to visit also becomes paramount [30, 31]. Lockdowns have been and will continue to be used as a risk management strategy to control the risk of cross infection in a community [6]. This study contributes to the growing evidence that residents of RACFs are vulnerable to the effects of restrictions on visitors and activities within RACF [32].

## Strengths, limitations, and recommendations for future research

The study took a population-based approach, rather than exploring individual changes pre- and mid-Covid-19. One reason was the access to a set of unique data sets, collected anonymously from residents in RACFs across Australia, which precluded the ability to link the data to individuals. Even though data were collected Australia wide, there was no representation from one state and two territories. Consistent with the population-based approach, the large secondary data set across multiple RACFs in different states created an inability to fully describe the sample (i.e. age; functional status; time living in the RACF) and details of the context in which they lived, which could be considered a limitation. Even though there was evidence of continued restrictions during the time of mid-covid-19 data collection [23], it cannot be ascertained specifically when lockdowns occurred in RACFs, which is also a limitation. However, if the aged care sector is to recommend or implement widespread measures to minimise the effect of lockdowns and restrictions, then an understanding of the effects across the population is required.

Another limitation of the population-based approach was the inability to probe further with respondents about the meaning behind their free text responses. To manage this limitation there was rigour applied in interpretation and organisation of the data through having multiple data analysts that regularly conferred (researcher triangulation) and reflexively discussed how to assign codes [33]. Codes stayed very close to words used by respondents and the final decisions about organising the codes into categories were made through consensus. An inherent assumption is made that the frequency of responses equates to perceived importance which may not necessarily reflect the truth. Finally, it is acknowledged that information on the cognitive status of the individual resident was not collected. Whilst this is a limitation of the study there is no reason to assume that the cognitive profile across both cohorts would have been significantly different. Further in-depth qualitative work is recommended that explores specific effects of restrictions and lockdown on the codes identified in this study, and perceptions about their relative importance. More specifically, there is scope for further exploration of ways to promote feelings of connectedness, general wellbeing and contentment, and the value of lifestyle activities during times where there are restrictions in place.

## Implications and conclusions

People living in RACFs completed the Happy Life Index at time points prior to and during the covid-19 pandemic. Responses to two open questions on the survey were analysed and compared across the two time points. It was found that residents noticed changes in staffing levels and visitors during periods of restrictions. During these times, residents appreciated the quality of the staff attending to their needs and the quality of their food. The categories and codes provide useful insight into some parameters and priorities for quality of life for residents in RACFs that may influence further development of residential aged care specific quality of life measures [34]. Currently in Australia, the mandatory quality indicators for aged care have a strong clinical focus (i.e. medication management and pressure care) [35]. The plans to increase these indicators to have a greater focus on the quality of life and consumer experience are currently under consultation. The findings from this research using HLI may contribute to this discourse. Furthermore, given the reporting of pre- and mid-covid-19 findings, managers, and policymakers can also use this information to make decisions about the cost–benefit of restrictions and strategies for continued sustainment of resident satisfaction despite the presence of risk mitigation lockdowns and restrictions.

## References

[1] Department of Health. (2014). National Framework for Communicable Disease Control.

[2] El Chakhtoura NG, Bonomo RA, Jump RLP (2017). Influence of aging and environment on presentation of infection in older adults. Infectious disease clinics of North America.

[3] Mitchell BG (2019). Organisation and governance of infection prevention and control in Australian residential aged care facilities: A national survey. Infection, Disease & Health.

[4] Garibaldi RA (1999). Residential care and the elderly: The burden of infection. Journal of Hospital Infection.

[5] Australian Institute of Health and Welfare. (2022). *Residential aged care facility: Identifying and definitional attributes*. METEOR online registry. Retrieved from December 9, 2022, from https://meteor.aihw.gov.au/content/384424

[6] Usher, K., et al. (2021). Preparedness for viral respiratory infection pandemic in residential aged care facilities: A review of the literature to inform post-COVID-19 response. *Journal of Clinical Nursing.* 10.1111/jocn.15863 PMC8242770 34021650

[7] Prime Minister of Australia. Update on Coronavirus measures—media statement. Retrieved March 18, 2020; February 28, 2022 from https://www.pm.gov.au/media/update-coronavirus-measures

[8] Chan DKY, McLaws ML, Forsyth DR (2021). COVID-19 in aged care homes: A comparison of effects initial government policies had in the UK (primarily focussing on England) and Australia during the first wave. International Journal for Quality in Health Care.

[9] Wu B (2020). Social isolation and loneliness among older adults in the context of COVID-19: A global challenge. Global Health Research and Policy.

[10] Royal Commission into Aged Care Quality and Safety, Aged care and covid-19: Special report. September 2020. Retrieved December 12, 2022, from https://agedcare.royalcommission.gov.au/publications/aged-care-and-covid-19-special-report

[11] Siette J (2021). The impact of COVID-19 on the quality of life of older adults receiving community-based aged care. Australasian Journal on Ageing.

[12] Sepúlveda-Loyola W (2020). Impact of social isolation due to COVID-19 on health in older people: Mental and physical effects and recommendations. The Journal of Nutrition, Health & Aging.

[13] Daly JR (2021). Health impacts of the stay-at-home order on community-dwelling older adults and how technologies may help: Focus group study. JMIR Aging.

[14] Brooks SK (2020). The psychological impact of quarantine and how to reduce it: A rapid review of the evidence. The Lancet.

[15] Siette J (2021). A national survey on COVID-19 second-wave lockdowns on older adults’ mental wellbeing, health-seeking behaviours, and social outcomes across Australia. BMC Geriatrics.

[16] van Dyck LI (2020). Combating heightened social isolation of nursing home elders: The telephone outreach in the COVID-19 outbreak program. The American Journal of Geriatric Psychiatry.

[17] Kasar KS, Karaman E (2021). Life in lockdown: Social isolation, loneliness and quality of life in the elderly during the COVİD-19 pandemic: A scoping review. Geriatric Nursing.

[18] Magennis T, Chenoweth L (2009). How can we improve residents' quality of life? Assessing the value and practicality of routine quality of life measurement in a residential aged care facility. Geriaction.

[19] Johs-Artisensi JL, Hansen KE, Olson DM (2020). Qualitative analyses of nursing home resident’s quality of life from multiple stakeholders’ perspectives. Quality of Life Research.

[20] DePoy E, Gitlin LN (2020). Introduction to research: Understanding and applying multiple strategies.

[21] Leung DY, Chung BPM, Liamputtong P (2019). Content analysis: using critical realism to extend its utility. Handbook of research methods in health social sciences.

[22] CarePage. (2022). *Happy Life Index: Measuring the moments that matter to older adults*. Retrieved 12 December 2022 https://happylifeindex.com.au/

[23] Commission., A.C.Q.a.S. (2021). *Residential care visitor access survey report*.

[24] Microsoft Corporation. Microsoft Excel Software. Retrieved from March 4, 2022, from https://www.microsoft.com/en-us/about

[25] Walker H, Paliadelis P (2016). Older peoples’ experiences of living in a residential aged care facility in Australia. Australasian Journal on Ageing.

[26] Milte R (2018). What characteristics of nursing homes are most valued by consumers? A discrete choice experiment with residents and family members. Value in Health.

[27] Henderson J (2018). The impact of facility ownership on nurses’ and care workers’ perceptions of missed care in Australian residential aged care. Australian Journal of Social Issues.

[28] Henderson J (2017). Missed care in residential aged care in Australia: An exploratory study. Collegian.

[29] Kelly, C. (2022). *A sector in crisis: Covid special report*. Australian Ageing Agenda (pp. 10–11) January–February.

[30] Gunn KM (2021). Choosing and managing aged care services from afar: What matters to Australian long-distance caregivers. International Journal of Environmental Research and Public Health.

[31] O'Caoimh R (2020). Psychosocial impact of COVID-19 nursing home restrictions on visitors of residents with cognitive impairment: A cross-sectional study as part of the Engaging Remotely in Care (ERiC) project. Frontiers in Psychiatry.

[32] Brydon A (2021). National survey on the impact of COVID-19 on the Mental Health of Australian Residential Aged Care Residents and Staff. Clinical Gerontologist.

[33] Liamputtong P (2012). Qualitative research methods.

[34] Courtney M (2003). Quality of life measures for residents of aged care facilities: A literature review. Australasian Journal on Ageing.

[35] Aged Care and Quality Safety Commission. (2022). National Aged Care Mandatory Quality Indicator Program. Retrieved from December 12, 2022, from https://www.agedcarequality.gov.au/providers/national-aged-care-mandatory-quality-indicator-program

